# Comparison of nonsurgical treatment approaches for partial rotator cuff tears: a systematic review and meta-analysis

**DOI:** 10.1016/j.xrrt.2026.100782

**Published:** 2026-05-19

**Authors:** Elena Ricker, Lara Stehling, Lisa Klute, Leopold Henßler, Helge Knüttel, Florian Zeman, Volker Alt, Maximilian Kerschbaum

**Affiliations:** a Clinic of Trauma Surgery, Regensburg University Hospital, Regensburg, Germany; b Medical Branch Library, University Library, University of Regensburg, Regensburg, Germany; c Center for Clinical Studies, Regensburg University Hospital, Regensburg, Germany

**Keywords:** Rotator cuff, Partial thickness rotator cuff tear, Injection therapy, Physical therapy, Partial rotator cuff tear, Non-surgical treatment, Meta-analysis, Systematic review

## Abstract

**Background:**

Rotator cuff disease constitutes the most common cause of shoulder pain, accounting for up to 70% of shoulder-related complaints, with partial-thickness rotator cuff tears (PT-RCTs) representing a substantial proportion. Although nonsurgical interventions are generally recommended as first-line treatment, there is currently no consensus on the optimal nonsurgical management of symptomatic PT-RCTs. This study aims to determine the effectiveness of different nonsurgical treatment modalities for PT-RCTs, specifically comparing physical therapy (PT) and injection therapies.

**Methods:**

A systematic review following Preferred Reporting Items for Systematic Reviews and Meta-Analyses (PRISMA) guidelines was performed using MEDLINE, EMBASE, Cochrane Central Register of Controlled Trials, ClinicalTrials.gov, and World Health Organization International Clinical Trials Registry Platform. Eligible studies on nonsurgical interventions for PT-RCTs in adults (≥18a) were included. Clinical outcomes (Constant Score (CS), American Shoulder and Elbow Surgeon (ASES) score, and visual analog scale were analyzed using random-effects meta-analysis and descriptive statistics to outline study characteristics.

**Results:**

From 9,894 records screened, 22 studies with 1,137 patients were included. PT was statistically superior to injection therapy in the CS (*P* = .0001). Among injections, cell-based therapies outperformed non–cell-based injections in the CS (*P* = .01) and exceeded Minimally Clinically Important Difference (MCID) for both the CS and ASES, indicating both statistical and clinical significance. Non–cell-based injections and PT show statistical significance in the CS (*P* < .001), with intervention groups outperforming control groups. Non–cell-based injections also surpass the clinical threshold in the CS (mean difference = 19.11), showing both statistical and clinical significance.

**Conclusion:**

In PT-RCTs, PT shows statistically superior outcomes to injection therapy in the CS. Cell-based injections outperform non–cell-based injections in the CS and exceed MCID in both, the CS and ASES scores, indicating clinical relevance. Both experimental groups for non–cell-based injections and PT show statistical significance in the CS, with non–cell-based injections surpassing the MCID in the CS. These findings highlight the effectiveness of PT and cell-based injections as nonsurgical options, but treatment should be individualized, as no single approach proved clearly superior across all outcomes.

Musculoskeletal disorders of the shoulder, particularly rotator cuff disease (RCD), pose a substantial clinical and societal challenge due to their high prevalence and significant impact on quality of life. RCD is the most common shoulder condition, accounting for up to 70% of shoulder-related medical consultations.^42^ The resulting limitations in daily functioning and well-being are considerable, with studies showing a reduction in quality of life comparable to that observed in chronic conditions such as diabetes.^15^ With the demographic trend toward an aging population, the prevalence of RCD is expected to increase further, placing an increasing burden on healthcare systems and affected individuals.^18^

Partial-thickness rotator cuff tears (PT-RCTs) represent a substantial proportion of RCD, with reported incidences ranging from approximately 17%^40^ to 40% in overhead athletes, specifically affecting the dominant shoulder.^55^ While some tears result from acute trauma, most develop through degenerative, age-related changes in tendon structure. The pathophysiology is multifactorial, involving complex interactions between tendon, bone, and muscle.^4^ Compared with full-thickness (FT) RCTs, PT-RCTs are estimated to be twice as prevalent and may progressively worsen, potentially resulting in FT-RCTs, greater functional impairment, and an increased need for medical intervention.^14^

Management strategies are broadly divided into surgical and nonsurgical options, with nonsurgical interventions typically recommended as first-line treatment.^35^ For the purpose of this study, these include structured physical therapy (PT), comprising exercises-based programs, trigger point treatments, and soft-tissue techniques, as well as injection-based therapies. Injections can be further categorized as cell-based options (platelet rich plasma [PRP], Uncultured, Autologous, Fresh, Unmodified, Adipose Derived Regenerative Cells [UA-ADRCs], adipose-derived mesenchymal stem cells [Ad-MSC]) and non–cell-based agents (corticosteroids, collagen, hyaluronate).

The increasing relevance of nonsurgical interventions is supported by meta-analyses, particularly for FT-RCTs, demonstrating their potential to reduce surgical risks, shorten rehabilitation periods, and lower healthcare costs.^3^ However, evidence for PT-RCTs remains fragmented. Existing reviews^32^ have mainly addressed individual modalities, such as PT or injection, rather than providing a direct comparison between them.

Due to the considerable heterogeneity and methodological differences among available studies, no definitive guidance exists regarding nonsurgical treatment for symptomatic PT-RCTs.

The aim of this systematic review and meta-analysis is therefore to critically evaluate the highest quality clinical evidence currently available and to provide a comprehensive comparison of nonsurgical treatment options for PT-RCTs.

## Methods

The protocol of this systematic review was prospectively registered in PROSPERO (ID: CRD42024588706) and is reported according to the Preferred Reporting Items for Systematic Reviews and Meta-Analyses (PRISMA) 2020, Preferred Reporting Items for Systematic reviews and Meta-Analyses literature search extension (PRISMA-S), and Terminology, Application, and Reporting of Citation Searching (TARCiS) guidelines.^19^^,^^41^^,^^43^

It was developed in reference to another registered protocol (ID: CRD42023487714), during the development of which a need to further investigate nonsurgical treatment options was identified. This review and meta-analysis therefore serve as a complementary investigation focusing specifically on nonsurgical treatment strategies.

### Inclusion and exclusion criteria

Initially, the decision was made to consider publications in both, English and German. However, in the final selection, only English-language studies were included, as the German texts were excluded based on other predefined exclusion criteria. The selection of studies for this review was based on predefined eligibility criteria: only studies involving adult participants aged 18 years or older with a confirmed diagnosis of PT-RCTs were considered. This diagnosis had to be established through physical examination, clinical assessment, or imaging modalities such as magnetic resonance imaging, arthrogram, or ultrasound. Studies were excluded if the inclusion and exclusion criteria for participant selection were insufficiently described, preventing a proper evaluation of whether the study population met the defined standards. Furthermore, studies conducted on animals, cadaveric specimens, or in vitro models were excluded from this review.

**Figure 1:**
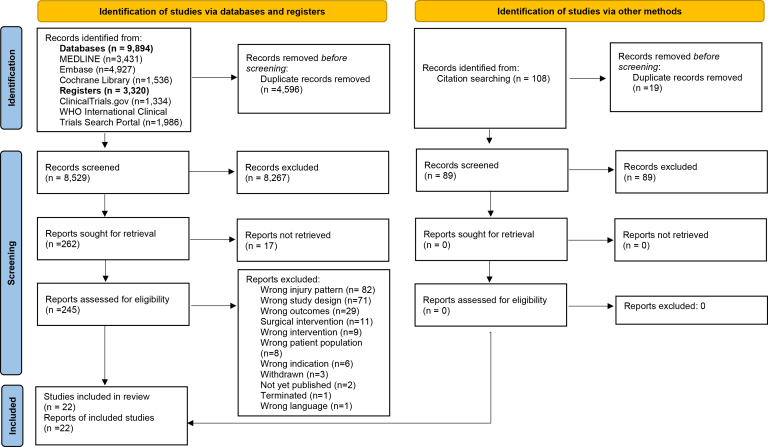
PRISMA flow diagram.^41^*PRISMA*, Preferred Reporting Items for Systematic Reviews and Meta-Analyses; *WHO*, World Health Organization.

The specific reasons for excluding studies during the full-text review phase are presented in the PRISMA flow diagram (Fig. 1), providing a transparent overview of the selection process.

### Study identification

The following databases and registers were searched in January 2024:^25^^,^^26^ MEDLINE (via Ovid), EMBASE (via Ovid), Cochrane Central Register of Controlled Trials (via Cochrane Library), Clinicaltrials.gov, World Health Organization International Clinical Trials Registry Platform search portal (http://apps.who.int/trialsearch/). Broad and comprehensive search strategies for MEDLINE and Embase were based on those used in recent Cochrane reviews. These strategies were then adapted to the other sources. The search strategies were comprised of a single concept “Population: Partial rotator cuff tears.” In the bibliographic databases MEDLINE and Embase, search filters were applied to restrict to randomized controlled clinical trials. No other filters were used during searching such as by date or language. As no intervention concept was used, the search targeted studies comparing surgical and nonsurgical treatment options, as well as studies evaluating these approaches independently.

As a search method supplementary to the database searches, we performed backward citation searching of all included studies in Lens.org using the CitationChaser Shiny app.^10^ If seed references were not indexed in Lens.org, we manually checked the seed references' reference lists. We iteratively repeated backward citation searching on newly identified eligible references until no further eligible references could be identified. Full search strategies and checklists are available in the public repository (see Data Availability Statement).

Records from the database searches were imported into Endnote software (version 20.6) for partial deduplication with the method of Bramer et al^5^ (semiautomatic steps A-C) and by database accession numbers. The records were then imported into Covidence^52^ online systematic review software, which employes automatic deduplication. Records from the citation searches were imported directly into Covidence.

### Screening and data collection

Screening of records and data collection were conducted using Covidence software. Abstract screening of all identified studies was independently conducted by 2 reviewers. Subsequently, the same reviewers assessed the full-text articles according to the predefined eligibility criteria. To determine eligibility for each synthesis, the intervention characteristics of each study were extracted and compared against the predefined criteria to ensure they matched the planned groups. In cases of disagreement, consensus was achieved through discussion with a third reviewer. The studies that met the inclusion criteria were then analyzed in detail by both authors, who systematically extracted data on general study characteristics, including study design, level of evidence, patient demographics, types of interventions, treatment modalities, and reported outcomes.

A particular focus of this review was the detailed analysis of the specific interventions applied in each study to enable a structured comparison of the different therapeutic approaches. Outcome data were collected based on the follow-up periods specified in each study and included the following evaluation scores: the Constant Score (CS), the American Shoulder and Elbow Surgeon (ASES) score, and the visual analog scale (VAS).

In 5 studies (Chun et al.,^9^ Godek et al.,^17^ Hurd et al.,^21^ Jo et al,^24^ Lundeen et al^33^), the outcome values had to be extracted from graphs or charts, whereas all other studies provided numerical outcome data directly in the text or tables.

### Data analysis

All statistical analyses were conducted using R^44^ (RStudio Version 2024.09.1 [Build 394]; R Studio, Posit, Boston, MA, USA) employing the packages ‘meta’ and ‘flextable.’ Two methods were applied to estimate effect sizes: the mean difference (MD) was used for detailed comparisons between groups, while the weighted mean (WM) was employed to analyze the overall distribution of mean scores. This dual approach allowed for the comparison of various nonsurgical treatment interventions.

For analyses based on MD, heterogeneity was evaluated using I^2^, τ^2^, and *P* values, while the overall effect was determined by calculating the Z-value along with its corresponding *P* value. In the case of WM analyses, heterogeneity was assessed through I^2^, τ^2^, and χ^2^, with χ^2^ statistics and *P* values also used to perform subgroup comparisons.

The I^2^ statistic was used to interpret heterogeneity, with the following definitions: 0-40% (low), 30-60% (moderate), 50-90% (substantial), and 75-100% (considerable). To evaluate heterogeneity and summarize estimates, forest plots were created. In the presence of substantial or considerable heterogeneity, a random-effects model was utilized to adjust for variability across studies. A *P* value of less than .05 was considered statistically significant. When data were insufficient for meta-analysis, descriptive statistics were applied to report outcomes.

## Results

### Study selection

The database searches identified a total of 14,490 records. After removing duplicates, 9,894 studies remained and were subjected to title and abstract screening. Of these, 245 studies were considered relevant and assessed in full-text review. Among the 245 full-text articles, 223 were excluded for not fulfilling the predefined eligibility criteria, with detailed reasons for exclusion presented in Figure 1. In addition, a citation search was conducted; however, none of the 89 additional records identified met the predefined eligibility criteria.

**Figure 2:**
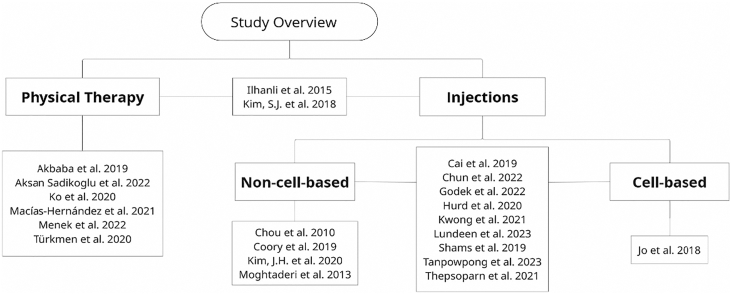
Study overview.

To obtain missing numerical outcome data or clarify key study details, we contacted the corresponding authors or study sponsors only, when necessary, with a 14-day interval between attempts. This was particularly relevant for studies available only as abstracts or those lacking complete participant data. Despite these efforts, 17 studies had to be excluded due to insufficient information, leaving 22 studies that met all eligibility criteria and were included in the final analysis (Fig. 2).

### Physical therapy vs. injection

There were 2 studies with a total of 94 patients, each group comprising 47 patients. The study by Ilhanli et al^22^ compared PRP injections with a variety of physiotherapeutic interventions, such as standard PT [hot pack for 15 minutes, ultrasound in continuous mode (1.5 W/cm^2^ for 5 min), transcutaneous electrical nerve stimulation (TENS) in brief-intense mode for 15 minutes, range-of-motion exercises, pandicular responses, stretching and strengthening exercises with 10 repetitions, as well as 500 mg acetaminophen as rescue medication], which was applied to the PT group for 15 sessions (5 sessions per week for three weeks). After the PT, the exercise program was continued as homework during the follow-up period. Classified as Level II evidence,^39^ the study utilized VAS scores and reported a significant improvement in rest-related VAS scores (*P* = .045), with the PT group showing superiority, whereas no significant differences were observed for VAS activity (*P* = .798) or sleep VAS (*P* = .222).

Kim, S.J. et al,^28^ a Level III Evidence study examined bone marrow aspirate PRP compared to exercise therapy. This Level III evidence study assessed outcomes using the ASES and VAS scores, demonstrating a statistically significant improvement in the bone marrow aspirate PRP group after three months (ASES: *P* = .011, VAS: *P* = .039) compared to the manual therapy group.

### Physical therapy

There were 6 studies with a total of 236 patients, all of which are RCTs providing Level II evidence. The studies conducted by Akbaba et al.,^1^ Aksan Sadigoglu et al,^2^ and Türkmen et al^50^ report both, ASES and VAS scores. Türkmen et al reported a statistically significant intragroup improvement (*P* < .0001) in pre- and post-therapy comparisons, but no significant differences were observed between groups. Similarly, Akbaba and Aksan Sadigoklu indicate that their results did not reach statistical significance.

In contrast, the studies conducted by Ko et al^29^ and Macias Hernandez et al^34^ reported only the CS and VAS scores. Ko et al, in a comparison of extracorporeal shock wave therapy with sham therapy, found a statistically significant improvement in both, CS (*P* = .005) and VAS (*P* = .025), demonstrating the superiority of shock wave therapy over the control treatment. Macias Hernandez et al also reported a significant intragroup improvement (CS, *P* = .01; VAS, *P* = .038), but no statistically significant differences between groups (CS, *P* = .654, VAS, *P* = .83). Finally, the study conducted by Menek et al^37^ assessed only the VAS score. Their study showed statistically significant improvement in pre- and post-therapy comparisons (*P* = .001), yet no intergroup significance was observed (*P* ≥ .05).

### Injection: non–cell-based vs. cell-based

There were 9 studies with a total of 536 patients, of whom 250 received therapeutic injections and 286 underwent cell-based therapy. All studies are RCTs, and thus are classified as Level II evidence. Cai et al^7^ assessed all 3 outcome scores (CS, ASES, VAS) and found PRP to be significantly superior compared to normal saline and sodium hyaluronate (SH) in various combinations (*P* ≤ .05). Among studies that employed both, ASES and VAS, Hurd et al^21^ demonstrated the superiority of UA-ADRCs over corticosteroid injection (CSI) (*P* < .05). In contrast Lundeen et al^33^ investigated the same comparison but did not observe any significant differences. Meanwhile, Kwong et al^31^ found PRP to be more effective than CSI (*P* = .03). Similarly, Shams et al^46^ and Tanpowpong et al,^47^ reported PRP to be superior to CSI at the twelve week follow-up based on CS and ASES scores. In Shams et al, the differences were highly significant (*P* < .001 for both CS and ASES), while Tanpowpong et al also observed significant improvements (CS: *P* = .02, ASES: *P* = .002).

Conversely, Chun et al^9^ found no statistically significant differences in ASES scores, while Thepsoparn et al^49^ and Godek et al^17^ used VAS to assess outcomes. Thepsoparn et al conducted a comparison between PRP and CSI, reporting a statistically significant advantage for PRP at the 6-month follow-up (*P* < .01). In contrast, Godek et al, who examined the effects of collagen and PRP individually as well as in combination, found no significant differences between treatment groups (VAS, *P* = .35).

The findings of Cai, Kwong, Shams, Tanpowpong, and Thepsoparn support the superiority of PRP over CSI, while Hurd et al demonstrate the effectiveness of UA-ADRCs. Collectively, these results suggest that cell-based therapy is the more effective treatment option in this context.

### Injection: non–cell-based

There were 4 studies with a total of 252 patients all of which were RCTs, providing Level II evidence. Three studies used both CS and VAS for data collection, while Kim, J.H. et al^27^ assessed all 3 outcome parameters (CS, ASES, VAS). In Kim's study, patients treated with Atelocollagen (n = 76) showed a statistically significant improvement (*P* ≤ .01) compared to those who did not receive Atelocollagen (n = 38).

Chou et al^8^ found no significant differences during the treatment period, but at the 6-week follow-up, a significant improvement in VAS scores was observed in the SH compared to placebo (*P* = .002). However, no such difference was observed in the CS (*P* = .091). Coory et al^11^ reported a statistically significant improvement in CS for patients treated with nerve block compared to subacromial injection (*P* = .003). Similarly, Moghtaderi et al^38^ found that SH was significantly more effective than placebo (*P* < .001).

### Injection: cell-based

It should be noted that there is one Level III evidence study focusing exclusively on cell-based therapy (Jo et al^24^). This study, conducted by Jo et al, includes 19 patients and examines the effects of different doses of AD MSC injections (1 × 10^7^, 5 × 10^7^, and 1 × 10^8^ cells) based on CS and VAS scores. The findings indicate that higher doses of AD MSCs are associated with significant improvements in outcome scores (*P* < .05).

### Methodological quality assessment

**Figure 3:**
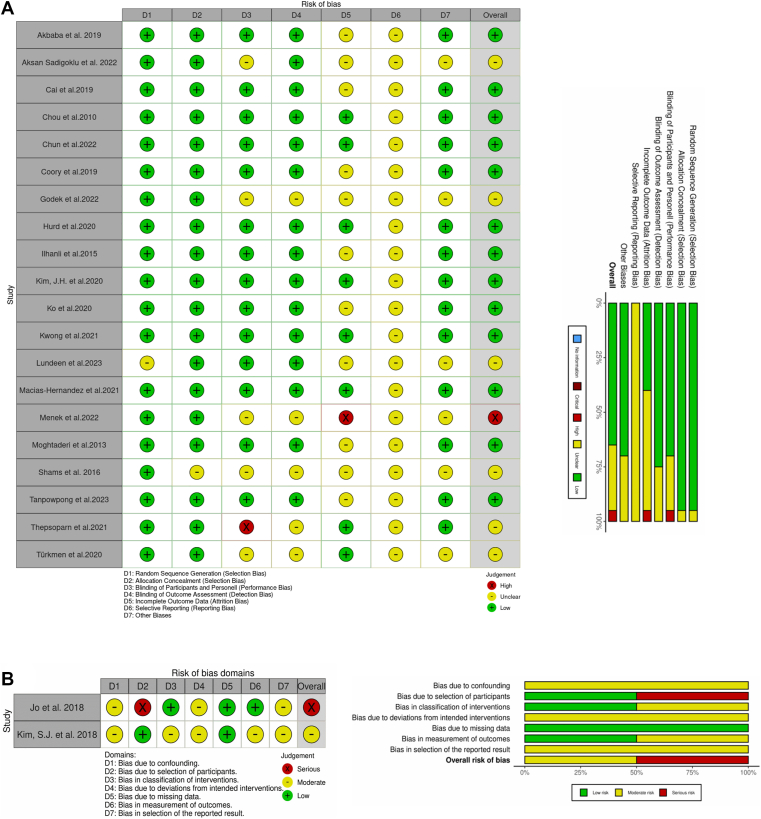
(**a**): Cochrane risk of bias RobGeneric tool for randomized studies; (**b**): Cochrane risk of bias ROBINS-I tool for nonrandomized studies.^36^*ROBINS-I*, Risk Of Bias In Non-randomized Studies - of Interventions.

The Cochrane Risk of Bias Tool for RCTs and non-randomized studies were used to evaluate the risk of bias in each included study. Given that 20 out of 22 studies are RCTs with a generally high standard of randomization, the selection bias results presented in Fig. 3a are consistent with expectations. The degree of performance and selection bias largely depend on the blinding methods used in each study, which are mostly single- or double-blind designs, leading to an overall low to moderate risk of bias in these domains. While attrition and detection bias are well-controlled in most studies, reporting bias remains a concern, potentially impacting the reliability of reported outcomes. As a result, the overall strength of evidence remains uncertain, as some studies demonstrate strict methodological control, whereas others present notable risks of bias that could affect the validity of their findings.

### Meta-analysis

Because of methodological differences and inconsistencies in the reported outcome measures among the available studies, only 17 studies could be included in the comparative analysis. Hurd et al was excluded due to missing standard deviation values. Furthermore, as the primary focus of this analysis was on the ASES and CS, the studies by Godek, Ilhanli, Menek, and Thepsoparn were not considered in the statistical evaluation, as they exclusively reported VAS outcomes.

## Discussion

**Figure 4:**
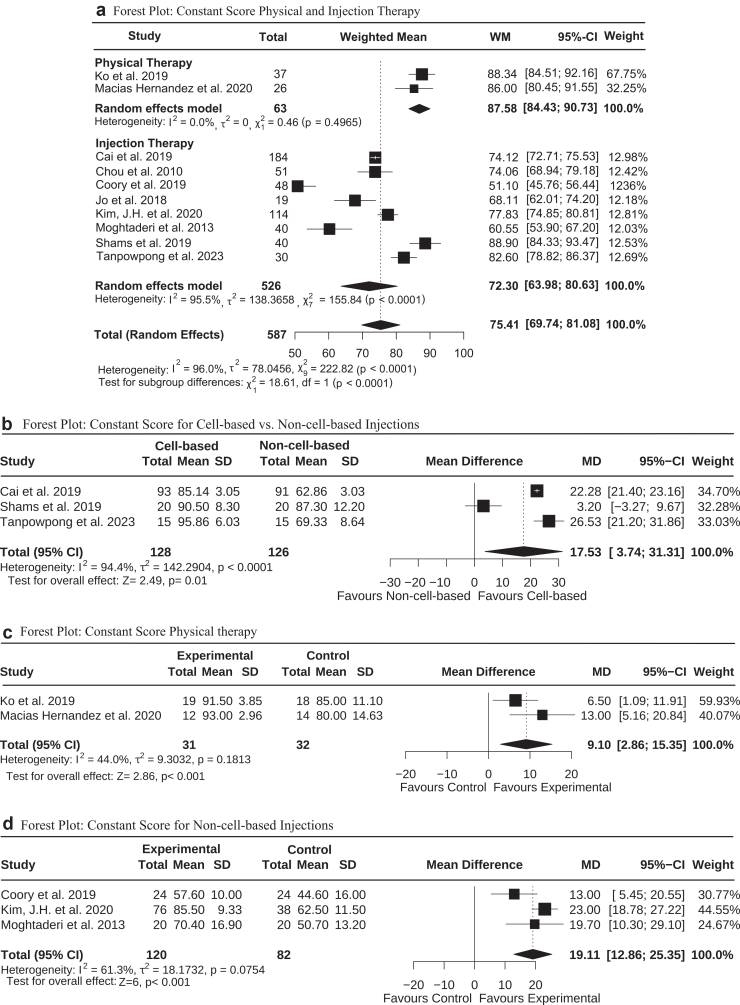
(**a-d**) Forest plots for CS between: a: physical and injection therapy; (**b**): cell-based vs. non–cell-based injection; (**c**) physical therapy vs. control; (**d**) non–cell-based therapy vs. control. *CI*, confidence interval; *SD*, standard deviation; *MD*, mean difference.

**Figure 5:**
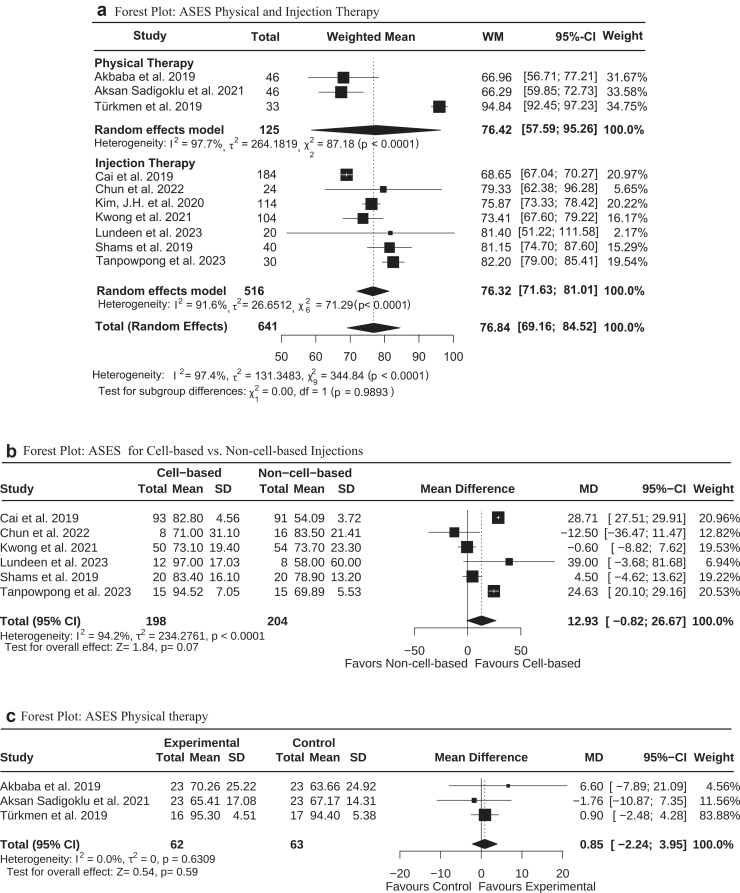
(**a-c**) Forest plots for ASES between: (**a**) physical and injection therapy; (**b**) cell-based vs. non–cell-based injection; (**c**) physical therapy vs. control. *ASES*, American Shoulder and Elbow Surgeons; *WM*, weighted mean; *CI*, confidence interval; *SD*, standard deviation; *MD*, mean difference.

The results indicate that, based on the comparison of WM, PT is statistically significantly superior to injection therapy in terms of the CS (*P* = .0001, Fig. 4*a*). However, no significant difference is observed for the ASES score (*P* = .9893, Fig. 5*a*). A closer examination of the MD between injection treatments reveals that cell-based injections demonstrate statistical superiority over non–cell-based injections for the CS (with *P* = .01, Fig. 4*b*), while no significant difference is observed for the ASES (*P* = .07, Fig. 5*b*). When analyzing non–cell-based injection therapies individually, statistical significance is observed in the CS (*P* < .001, Fig. 4*d*) for the experimental group. Similarly, various PT modalities demonstrate statistically significant differences in the CS (*P* < .001, Fig. 4*c*) but not in the ASES (*P* = .59, Fig. 5*c*).

When considering the Minimally Clinically Important Difference, which is crucial for determining clinical significance and relevance, the comparison of cell-based to non–cell-based therapies shows a MD of 17.53 points in the CS, exceeding the required threshold of 10.4 points.^30^ For the ASES score, the Minimally Clinically Important Difference range of 11-17 points^12^^,^^48^ is met with an MD of 12.93 points. Therefore, cell-based injection therapy demonstrates not only statistical significance in the CS but also clear clinical relevance in both, the CS and ASES. Non–cell-based therapies also achieve an MD of 19.11 points in the CS, surpassing the clinical threshold, thereby demonstrating both, statistical and clinical relevance.

These findings emphasize the important role of PT as a nonsurgical treatment option to improve shoulder function. Exercise therapy, a core element of PT, has been shown to be an effective first-line treatment for patients with FT-RCTs, significantly improving pain and function. Moreover, it may reduce the need for surgery, offering a valuable nonoperative alternative in selected cases.^13^ In addition to exercise therapy, electrotherapy modalities such as microcurrent electrical neuromuscular stimulation and transcutaneous electrical nerve stimulation have also demonstrated effectiveness. Vrouva et al^53^ reported that these techniques contribute to pain reduction, functional improvements, and enhanced quality of life in patients with PT-RCTs.

Within the field of injection therapy, particular attention is given to CSI, PRP, UA ADRCs, AD MSC, collagen, and hyaluronate. Among these, CSI is a well-established option for managing shoulder pathologies.^6^^,^^51^ Systematic reviews by De Sanctis et al^16^ and Zhu et al^55^ further support our findings, reporting superior outcomes associated with PRP injections. Notably, while CSI has been shown to be highly effective in reducing pain in the short and midterm, PRP demonstrates superior long-term outcomes, providing greater pain relief and functional enhancement.^16^ Furthermore, preclinical studies have shown that MSCs can reduce inflammation, promote tissue remodeling, and improve tendon strength. Early clinical trials further suggest potential benefits in lowering retear rates and enhancing functional outcomes.^20^

Current evidence suggests that nonsurgical therapies are not only effective as standalone treatments but also play a crucial role when combined with surgical interventions. They serve as an essential pretreatment strategy for PT-RCT management^32^ and are also applied in post-operative rehabilitation for shoulder conditions, as evidenced in studies on RCTs,^45^ reinforcing the importance of an integrated treatment approach.

Regarding the classification of PT-RCTs, Ellman's system categorizes these lesions based on location—articular, bursal, or intra-tendinous—and depth: less than 3 mm (<25%), 3-6 mm (25-50%), and greater than 6 mm (>50%).^23^ This classification holds clinical relevance, as research has shown that the severity of symptoms tends to increase with tear size, and even small asymptomatic tears may progress to irreparable lesions over time if left untreated.^54^ However, an analysis of the included studies reveals inconsistency regarding the type of PT-RCTs treated, contributing to variability in study populations and potentially influencing clinical outcomes.

One of the primary limitations of this meta-analysis is methodological and clinical heterogeneity among the included studies. Differences in study design, patient populations, treatment interventions, and the classification of studies into large comparison groups complicate result comparison and limit generalizability of the findings. In addition, it was not possible to extract from the included studies the exact mechanism of injuries, nor to consistently assess the severity and the precise localization of the tear. The substantial heterogeneity of injection therapies represents an important limitation of this meta-analysis, as the included interventions differ markedly in composition and biological mechanism. To allow a structured and clinically interpretable synthesis despite this heterogeneity, treatments were pragmatically classified into cell-based and non–cell-based approaches according to their predominant biological mode of action. Moreover, substantial statistical heterogeneity, reflected by high I^2^ values, further reduces the certainty of the pooled estimates. In addition, follow-up periods varied widely, making direct comparisons difficult and potentially under-representing long-term outcomes and side effects.

Another important limitation is the inclusion of 2 studies with an evidence level of III or lower alongside RCTs. Since lower-level evidence studies are more susceptible to bias, their inclusion may impact the overall reliability of the results.

Despite these limitations, this meta-analysis offers several key strengths. The thorough and systematic search approach, combined with the fact that over 90% of the included studies are RCTs, ensures a strong evidence base. In addition, the evaluation of 3 primary outcome measures allows for in-depth comparisons across different treatment approaches. By incorporating studies with comparable therapeutic strategies, this analysis facilitates meaningful insights not only in distinguishing injection therapy from PT but also in assessing variations within each treatment category.

In conclusion, although the limitations of this meta-analysis must be taken into account interpreting its results, it offers a valuable and comprehensive evaluation of the current evidence regarding the management of PT-RCTs. Nevertheless, larger studies with longer follow-up periods are needed to allow for a more precise assessment of treatment outcomes and long-term effects.

## Conclusion

This meta-analysis provides a comprehensive comparison of nonsurgical treatment approaches for PT-RCTs. While PT demonstrates statistical superiority over injection therapy in some outcome measures, its clinical significance remains uncertain. Among injection therapies, cell-based treatments appear more effective than non–cell-based options, with evidence suggesting both statistical and clinical relevance. However, these findings must be interpreted with caution due to the methodological and clinical heterogeneity, as well as variability in study quality. Overall, the results highlight the importance of individualized treatment approaches, considering the varying effectiveness of different nonsurgical strategies. Further high-quality RCTs with long-term follow-up are essential to refine treatment recommendations and assess the durability of therapeutic effects.

## Disclaimers:

Funding: No funding was disclosed by the authors.

Conflicts of interest: The authors, their immediate families, and any research foundations with which they are affiliated have not received any financial payments or other benefits from any commercial entity related to the subject of this article.

## Data availability

Data supporting this study is available from a public repository (DOI: 10.5283/epub.7942). This includes search strategies, accession numbers of the records found and PRISMA-S and TARCiS checklists for the search process, PRISMA 2020 checklist, list of excluded studies and a table of included studies.
